# The Role of Parvalbumin, Sarcoplasmatic Reticulum Calcium Pump Rate, Rates of Cross-Bridge Dynamics, and Ryanodine Receptor Calcium Current on Peripheral Muscle Fatigue: A Simulation Study

**DOI:** 10.1155/2016/3180205

**Published:** 2016-10-20

**Authors:** Oliver Röhrle, Verena Neumann, Thomas Heidlauf

**Affiliations:** ^1^Institute of Applied Mechanics (CE), University of Stuttgart, Stuttgart, Germany; ^2^Stuttgart Research Centre for Simulation Technology (SimTech), University of Stuttgart, Stuttgart, Germany

## Abstract

A biophysical model of the excitation-contraction pathway, which has previously been validated for slow-twitch and fast-twitch skeletal muscles, is employed to investigate key biophysical processes leading to peripheral muscle fatigue. Special emphasis hereby is on investigating how the model's original parameter sets can be interpolated such that realistic behaviour with respect to contraction time and fatigue progression can be obtained for a continuous distribution of the model's parameters across the muscle units, as found for the functional properties of muscles. The parameters are divided into 5 groups describing (i) the sarcoplasmatic reticulum calcium pump rate, (ii) the cross-bridge dynamics rates, (iii) the ryanodine receptor calcium current, (iv) the rates of binding of magnesium and calcium ions to parvalbumin and corresponding dissociations, and (v) the remaining processes. The simulations reveal that the first two parameter groups are sensitive to contraction time but not fatigue, the third parameter group affects both considered properties, and the fourth parameter group is only sensitive to fatigue progression. Hence, within the scope of the underlying model, further experimental studies should investigate parvalbumin dynamics and the ryanodine receptor calcium current to enhance the understanding of peripheral muscle fatigue.

## 1. Introduction

The motor units of a skeletal muscle range from the smallest motor unit (the motor neuron with the lowest excitation threshold innervating the muscle unit with the fewest and most slowly contracting muscle fibres) to the largest motor unit (the largest motor neuron innervating the muscle unit with the most and fastest contracting muscle fibres) [1]. Sustained muscle contractions at moderate to high force levels lead to fatigue and subsequently to a drop in exerted muscle force. Muscle fatigue can hereby be divided into central and peripheral fatigue [2]. Central fatigue refers to changes in the motor neuron activation pattern leading to a decrease in active force. Peripheral fatigue can be assigned to changes in the pathway from electrical excitation to contraction within the muscle fibres, which varies considerably among the different muscle units of a muscle [3]. For example, changes in the shape and amplitude of the action potential during sustained contractions have an influence on peripheral fatigue [4, 5]. The action potential is influenced by intracellular and extracellular sodium (Na^+^) and potassium (K^+^) concentrations as well as by membrane resistance and capacitance. Furthermore, accumulation of K^+^ and depletion of Na^+^ ions in the t-tubule system contribute to fatigue. Due to the small volume of the t-tubules, however, the exact concentration changes are difficult to determine experimentally. Other aspects, like the role of Ca^2+^, are undisputed. Failing to release Ca^2+^ from the sarcoplasmic reticulum (SR) causes fatigue. The Ca^2+^ concentration in the myoplasm is influenced by the ryanodine receptor (RyR) Ca^2+^ currents, the SR Ca^2+^-pump, and several Ca^2+^ buffers in the cell, such as parvalbumin, calmodulin, and ATP [6]. For repeated muscle contractions, the SR Ca^2+^-pumping and cross-bridge cycling increase the concentration of inorganic phosphate. Experiments suggest that high phosphate levels also play a key role in muscle fatigue.

The differences in the underlying biophysical processes of the different muscle units lead to variations in physiological behaviour such as in an alteration of the contraction speed, intracellular calcium (Ca^2+^) handling, or fatigue resistance [7]. While the differences in physiological behaviour are used to classify different muscle units, the expression of a part of the myosin molecules, the myosin heavy chain (MHC), is the most established classification for muscle units. The isoform of the MHC influences the rate of cross-bridge cycling and thus the maximal shortening velocity [8]. Based on the MHC classification, the major fibre types are types I, IIa, IIx, and IIb. This classification corresponds to the one developed in [3], distinguishing slow-contracting fibres (type S), fast-contracting fatigue-resistant fibres (type FR), and fast-contracting fatigable fibres (type FF) by a certain stimulation protocol (cf. [9]). This classification into discrete classes, however, does not apply to human motor units [10, 11]. There, a rather continuous variation from one extreme to another is observed for many functional properties [10, 12]. In fact, functional muscle properties such as contraction time, maximum shortening velocity, or the activity of the mitochondrial enzymes such as succinate dehydrogenase (SDH) show large and continuous ranges of variability across the motor units of human skeletal muscles [13].

The continuous variation of physiological properties within muscle units and the large number of different physiological quantities involved within the fatigue process challenge experimentalists to identify the key mechanisms contributing to fatigue. A detailed biochemical* in silico* model of force generation and fatigue in skeletal muscle can provide an analytical tool to analyse the sensitivity of muscle fatigue due to changes in individual processes or ion concentrations. Therefore, such a computational model could provide insights on the quantities playing a key role in muscle fatigue and hence support experimentalists in interpreting their findings.

As far as* in silico* models are concerned, there exists a large number of skeletal muscle models. However, many of these models either focus only on small parts of the excitation-contraction coupling (ECC) or phenomenologically describe the respective relationships and are therefore of less use in further investigating muscle fatigue. An exception is the biophysical model of the ECC published in [14]. This model unifies descriptions of membrane voltage [15], release of Ca^2+^ from the SR [16], activation of the cross-bridge cycling [17, 18], and the resorption of Ca^2+^ into the SR. Thus, it models all relevant processes of the ECC in a biophysical manner. The model includes aspects of muscle fatigue by assuming that inorganic phosphate, which is generated during cross-bridge cycling, is passively transported into the SR, where it can precipitate with Ca^2+^. As a consequence, less Ca^2+^ can be released from the SR and fewer cross-bridges can take part in the contraction yielding less force. Moreover, the Hodgkin-Huxley-type model of the membrane electrophysiology is capable of describing membrane fatigue by the accumulation of K^+^ and depletion of Na^+^ ions in the t-tubule system and the extracellular space.

In [14], two sets of parameters are provided: one for fast-twitch and one for slow-twitch muscles. Both parameter sets are based on literature values. The fast-twitch parameter set has been validated using experimental data obtained through investigating the effects of different electrical stimulation patterns on force production in mouse extensor digitorum longus (EDL) and the slow-twitch parameter set by doing similar experiments on the mouse soleus muscle. While these parameter sets represent extreme values, data for other muscle units are lacking.

To provide muscle unit specific parameters for investigating the key underlying physiological processes leading to fatigue, this work focuses on investigating different interpolation assumptions leading to individual parameter sets for distinct muscle unit types. Further, a sensitivity analysis is performed to investigate how the interpolated parameters, specifically the parameters associated with parvalbumin, the SR calcium pump rate, the cross-bridge cycling rates, and the RyR calcium current influence peripheral muscle fatigue. Note that it is not the aim of this study to identify an optimal set of parameters to model a specific muscle but rather to identify the parameters that significantly affect the considered functional measures, to investigate the sensitivity of the model to changes in the parameters, and to demonstrate that the approach of parameter interpolation is flexible enough to account for many different muscles.

## 2. Methods

### 2.1. Model Extension

This work is based on the biophysical model of the excitation-contraction coupling of Shorten et al. [14], which can be freely downloaded from the CellML website (https://www.cellml.org/). For the model, two distinct sets of parameters exist. Due to the fact that mouse EDL muscle consists almost exclusively of type-II fibres [19], the fast-twitch parameter set in [14] can be associated with a fast-twitch muscle unit. Similarly, soleus muscle consists mainly of type-I fibres [20], and hence the slow-twitch parameter set in [14] is associated with a slow-twitch muscle unit. These two sets of parameters are the basis for this work.

In the present work, the behaviour of a small human muscle consisting of 100 muscle units (e.g., a hand muscle, such as the first dorsal interosseous [10]), is investigated. To take into account that contraction times are longer in human than in mouse muscles [11, 14, 21], all parameters associated with cross-bridge cycling have been scaled by 1/10. Note that the rates obtained from this scaling for the slow-twitch version correspond to the parameters originally proposed by Razumova et al. for their cross-bridge dynamics model [18], which is used within [14]. The resulting contraction times of 93.7 ms and 30.5 ms for the slow-twitch muscle unit and the fast-twitch muscle unit, respectively, compare well to the values proposed in [11].

One drawback of the Shorten et al. model in the context of the current work, however, is the fact that the binding of calcium and magnesium ions to parvalbumin is modelled in a simplified way. In the model, the binding relies on the total amount of parvalbumin in the cell. This modelling assumption is sufficient if one intends to consider only type-I and type-II muscle units. In detail, following [22], Shorten et al. consider parvalbumin only in the fast-twitch version of their model, while the parvalbumin binding rates in the slow-twitch version are set to zero. Since the total parvalbumin concentration is not known for intermediate muscle units, the approach needs to be modified.

**Figure 1:**
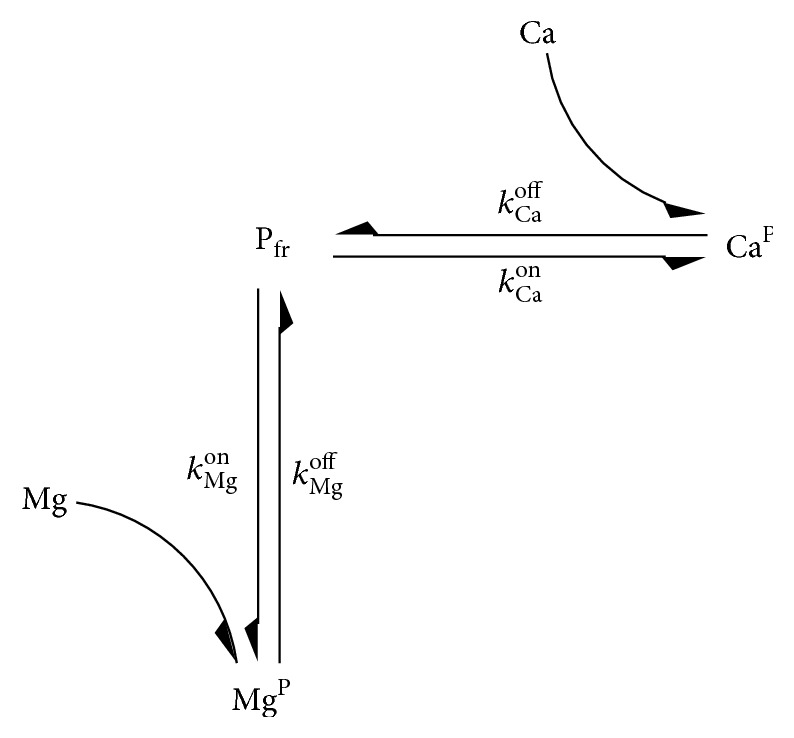
Chemical equilibrium model for parvalbumin.

The parameter representing the total parvalbumin concentration can be omitted if the process is modelled as described in the following. Parvalbumin can bind either to calcium or to magnesium. The respective binding rates are *k*_Ca_^on^ and *k*_Mg_^on^; the respective dissociation rates are *k*_Ca_^off^ and *k*_Mg_^off^. The reaction kinetics involving the concentration of free parvalbumin, P_fr_, are depicted in Figure 1 and are described by

(1) $$\begin{array}{c} \frac{dP_{fr}}{dt}=-k_{Ca}^{on}P_{fr}Ca-k_{Mg}^{on}P_{fr}Mg+k_{Ca}^{off}Ca^{P}+k_{Mg}^{off}Mg^{P}, \end{array}$$

where Ca represents the concentration of calcium ions, Mg is the concentration of magnesium ions, and Ca^P^ and Mg^P^ denote the parvalbumin bound calcium and the parvalbumin bound magnesium, respectively. Equation (1) is added to the general myoplasm and to the terminal SR myoplasm compartments. The parameter representing the concentration of free parvalbumin is then inserted into the equations of the model, where free parvalbumin is originally modelled by the difference of total parvalbumin concentration (constant) and the sum of the parvalbumin bound ion concentrations. This model extension now allows investigating different parameter sets for intermediate muscle units.

*Interpolation of Material Parameter Sets for Intermediate Muscle Units*. Currently, there exists no experimental study that could provide for this model a reasonable set of parameters to simulate intermediate muscle units. However, using* in silico* experiments, parameter sets for intermediate muscle units can be derived from the two extreme parameter sets, that is, from the parameter sets for slow-twitch and fast-twitch muscle units, through interpolation.

In the following, the slowest muscle unit is denoted by *α* = 0 and the fastest muscle unit by *α* = 1. Further, their corresponding parameter values are denoted by *μ*_0_ and *μ*_1_, respectively. Following [10, 11], this work assumes that the biophysical properties of muscle units are distributed continuously. Hence, the set of parameters for an intermediate muscle unit can be interpolated using a monotone continuous function. Here, the following interpolation functions are considered:

(2) $$\begin{array}{c} \mu \left(\;\alpha ,S\;\right)=\left(\;1-\alpha^{p_{i}}\;\right)\cdot \mu \left(\;0,S\;\right)+\alpha^{p_{i}}\cdot \mu \left(\;1,S\;\right), \end{array}$$

where *α* ∈ [0,1], *p*_*i*_ ∈ *ℝ* ^+^, *μ*(*α*, *S*) is the interpolation of parameter *S* for intermediate muscle unit *α*, and *p*_*i*_ denotes the exponent that characterises the curvature of the function interpolating the two fix points, that is, the polynomial interpolation of the respective slow-twitch (*α* = 0) and fast-twitch (*α* = 1) values, *μ*(0, *S*) and *μ*(1, *S*), respectively, of parameter *S*. Note, for *p*_*i*_ = 1, the respective parameters are interpolated linearly. For *p*_*i*_ > 1 the interpolated values remain for a wider range of *α* closer to the first value, *μ*(0, *S*), before accelerating to the second value, *μ*(1, *S*). For *p*_*i*_ < 1, the behaviour is just the opposite.

Within the extended model, there are a total of 31 parameters that vary from muscle unit to muscle unit (while 74 of the model's 105 parameters are similar in the slow-twitch and fast-twitch parametrisations). In order to manage this multitude of parameters, all of the 31 parameters are assigned to one of the following 5 groups. 

*Group 1* contains as only parameter the Ca^2+^ uptake rate into the SR. This parameter is expected to influence the contraction times. The rate of force generation decreases with increasing rate of the Ca^2+^-pump. 

*Group 2* groups the reaction rate constants of the cross-bridge cycle. They are expected to change the contraction times [24]. 

*Group 3* contains as only parameter the RyR Ca^2+^ current. Like the Ca^2+^ uptake rate, the RyR Ca^2+^ current is expected to influence the contraction times as the rate of force generation increases with the rate of Ca^2+^ release (RyR current). 

*Group 4* groups parameters that describe the binding to and the dissociation from parvalbumin of Ca^2+^ and magnesium (Mg^2+^) ions. Parvalbumin is the most important antagonist of troponin and thus influences the activation of the cross-bridge cycling. A change in fatigue characteristics is expected. 

*Group R* contains the rest of the parameters. These parameters are interpolated linearly by default.

**Figure 2:**
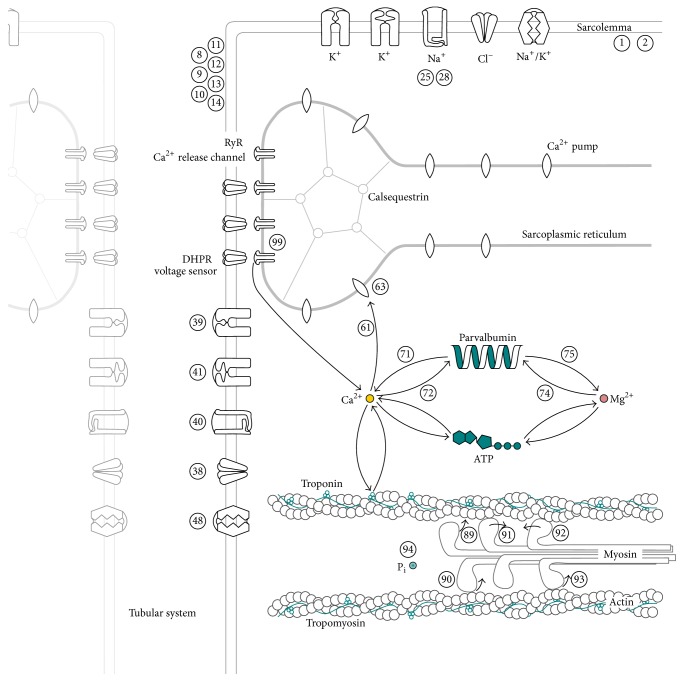
Schematic drawing of the cell model with variable parameters. The names of the parameters associated with the numbers within this drawing are given in Table 1.

Figure 2 depicts a schematic overview of the model with variable parameters. Each number indicates a parameter that has a different value for fast-twitch and slow-twitch muscle units and thus will be interpolated for intermediate muscle units.

The grouping of these parameters can be found in Table 1. By choosing for each group an individual interpolation parameter *p*_*i*_, the parameters associated with these groups can be interpolated individually.

### 2.2. Model Verification Approach

After choosing a particular interpolation, that is, choosing for each group in (2) a specific *p*_*i*_, the behaviour of the muscle units is compared to experimental data. Based on the data available from the literature, two characteristic force responses are considered for this purpose: (i) the contraction time (also referred to as time to peak) of the single twitch and (ii) fatigue progression.

The contraction time is defined as the duration of time between initiating a contractile response through a stimulus and the maximum twitch force response. Although each muscle has a specific frequency distribution of the muscle unit contraction times, histograms showing experimental data can be described as nearly Gaussian-shaped; see, for example, [13, 25–28].

For a quantitative evaluation of the simulation results, we use as “gold standard” a Gaussian (normal) distribution of the contraction times with 65 ± 10 ms (mean ± standard deviation). This leads to contraction times approximately between 40 ms and 90 ms (cf. [11]).

Fatigue progression is the development of the force under sustained tetanic stimulation. In [23], a 30 s force record for a fast-twitch muscle fibre stimulated at 70 Hz is presented. Taking [23] as a reference, the force of a type FF muscle unit (*α* = 1) after 5000 ms of sustained high-frequency stimulation should be about 70% to 75% of its maximum value. The force responses of fast fatigable, fast fatigue-resistant, and slow muscle units stimulated at 25 Hz for 1 s, which can be used as additional experimental data for fatigue progression, is presented in [3].

Furthermore, [3] defined a “fatigue index” by the ratio of the “maximum tension produced during the 120th tetanus (i.e., after 2 min of stimulation) to the tension output during the first tetanus in a standard sequence” (tetanic stimulation for 330 ms at 40 Hz repeated every second). This definition leads to the fact that slow muscle units without fatigue have a fatigue index of 1, while fatigable fast muscle units have a fatigue index of 0. According to [29], over 60% of the muscle units have a fatigue index greater than 0.75, that is, showing only little fatigue.

Due to high computational costs, the stimulation sequence in [3] is not simulated for all intermediate muscle units to determine the fatigue index. Instead, the fatigue index is determined from shorter stimulations. Nevertheless, the frequency distribution based on the fatigue index is adapted from the experimental results in [29]; that is, about 65% of the muscle units shall not show fatigue. Within this work, one assumes that a muscle unit shows no fatigue if the decline in force is less than 10% after continuously stimulating the muscle unit for 5000 ms with 100 Hz.

For a quantitative evaluation of the simulation results, we assumed as “gold standard” that the muscle units with *α* = {0,0.5,0.6,0.7} do not show fatigue after a continuous stimulation with 100 Hz for 5000 ms [29], and the force of the muscle unit with *α* = 1 is at 70% of its maximum value [23].

In summary, the verification of the suitability of a particular interpolation scheme describing the biophysical behaviour of the muscle units within the extended model is based on a quantitative comparison between the simulation output and the respective experimental data. In detail, for each combination of interpolation coefficients, the relative errors (2 norms of the relative differences between the simulation results and the above defined “gold standards”) are computed for the contraction times and the fatigue progression. Furthermore, the sensitivity of fatigue to changes in the exponent *p*_*i*_ within the interpolation scheme (2) provides the experimentalist with a simulation-based indication on potentially important mechanisms leading to peripheral muscle fatigue.

## 3. Results

First, the parameters of a specific muscle unit are determined through (2) by choosing a particular *α* (specifies the muscle unit) and by selecting for each parameter group a specific *p*_*i*_. The entire set of parameters is obtained for all 100 muscle units by uniformly sampling *α* ∈ [0,1]. To determine the contraction times for each parameter set and for each muscle unit, a single twitch is simulated. To compare the simulated contraction times with experimental studies, the contraction times are depicted within a histogram containing 15 bins. For the fatigue analysis, the force responses of 5 different muscle units are simulated, that is, for *α* = {0,0.5,0.6,0.7,1}, using a stimulation protocol that continuously excites the cellular model at a frequency of 100 Hz. The stimulation starts after 100 ms and lasts up to 5000 ms.

The starting point for investigating the fatigue behaviour of intermediate muscle units are simulations, in which the parameter sets are determined through a uniform interpolation; that is, all *p*_*i*_ are equal. Subsequently, the parameters in Groups 1–4 are varied by choosing within (2) individual exponents for each parameter group; that is, **p** = [*p*_1_, *p*_2_, *p*_3_, *p*_4_, *p*_*R*_], where the subindices refer to the group numbering. Unless otherwise stated, the parameters of Group *R* are linearly interpolated. For each chosen parameter set, the contraction times histogram and selected motor unit force responses at 100 Hz stimulation frequency are shown.

The relative errors between selected simulation results and the “gold standards” with respect to the contraction times and the fatigue progression are summarized in Table 2. Note that more simulations have been performed, but the chosen selection is sufficient to indicate the sensitivity of the model to changes in the interpolation coefficients. In detail, if relative small changes in an interpolation parameter led to significant changes in the functional measures, we restrained from displaying further data resulting from simulations where larger variations in the interpolation coefficients have been applied. Due to the fact that similar errors can arise from very different distributions, detailed information on the distribution of the contraction times and the fatigue progression are provided in the following (cf. Figures 3 [Fig fig4] [Fig fig5] [Fig fig6] [Fig fig7] [Fig fig8] [Fig fig9] [Fig fig10] [Fig fig11]–12 and Supplementary Figures  17–38; see Supplementary Material available online at http://dx.doi.org/10.1155/2016/3180205).

Supplementary Figures 17 and 18 show the above-mentioned relations for a linear interpolation of all parameters; that is, **p** = [1,1, 1,1, 1]. In addition, other uniform interpolations for all parameters are investigated. Figure 19/Figure 20, Figure 21/Figure 22, and Figure 23/Figure 24 within the Supplementary Material show the results for **p** = [2,2, 2,2, 2], **p** = [5,5, 5,5, 5], and **p** = [0.5,0.5,0.5,0.5,0.5], respectively. In all these simulations, intermediate muscle units are more affected by fatigue than the fastest muscle unit (*α* = 1). This behaviour does not agree with experimental data. Since the slowest muscle unit (*α* = 0) shows no fatigue and the parameters associated with the binding to and dissociation from parvalbumin are uniformly equal to zero for this muscle unit, it is concluded that the parameters of the parvalbumin group have to be modified first.

### 3.1. Sensitivity with respect to Parvalbumin Parameters

First, the fast-twitch parameter set is fitted such that the fastest muscle unit (*α* = 1) exhibits 70% of its maximal force after stimulating the muscle unit for 5000 ms with a 100 Hz frequency. This result can only be obtained when the parameters in the parvalbumin group (Group 4) are scaled by 1/20. (The parameters of the parvalbumin group for the slow-twitch parameter set are all equal to zero.)

**Figure 3:**
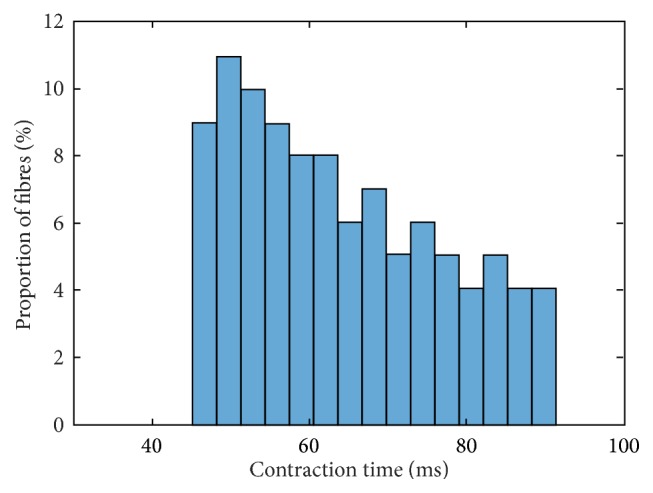
Frequency distribution of contraction times based on a linear interpolation of all parameters; that is, **p** = [1,1, 1,1, 1].

**Figure 4 fig4:**
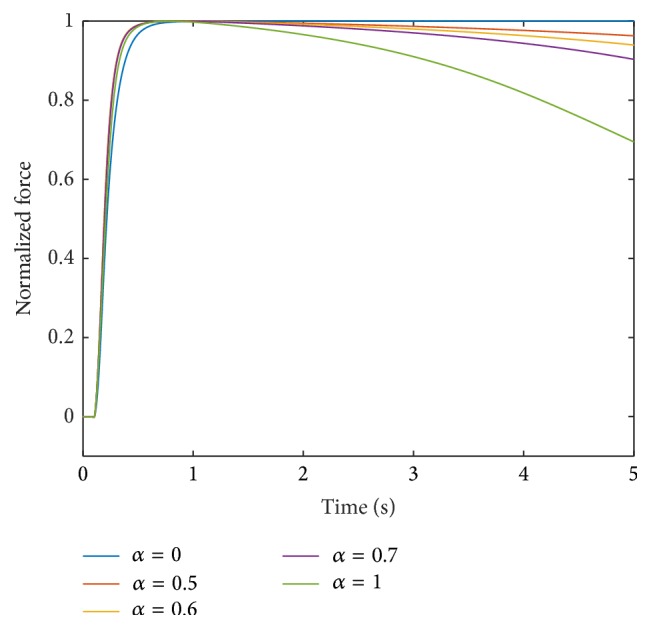
Force response at 100 Hz stimulation for different muscle units obtained through linear interpolation of all parameters; that is, **p** = [1,1, 1,1, 1].

Incorporating this scaling, the contraction times and force responses using a linear interpolation for all parameters, that is, **p** = [1,1, 1,1, 1], are depicted for intermediate muscle units in Figures 3 and 4, respectively.

The contraction times are partially higher compared to the original parvalbumin parameter set (cf. Figures 17 and 18 within the Supplementary Material). They range from 45 ms to 91 ms. Particularly, the fast-twitch muscle units exhibit reduced contraction times if compared to the original parvalbumin parameter set. As a consequence, the histogram is less steep.

The force of the fastest muscle unit after 5000 ms is 70% of its maximum value. All other muscle units show less fatigue than the muscle unit with *α* = 1. The muscle units with *α* = 0.5, 0.6, and 0.7 are at 96%, 94%, and 90% of their maximum forces, respectively, after 5000 ms.

### 3.2. Sensitivity with respect to Calcium Pump Uptake Rate

**Figure 5 fig5:**
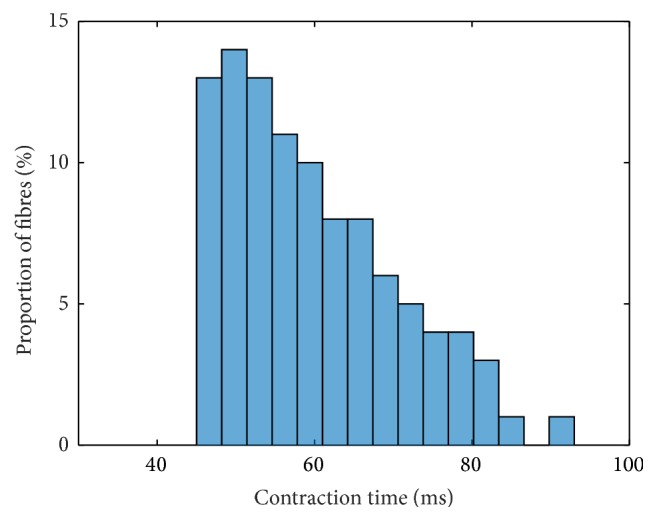
Frequency distribution of contraction times based on choosing for interpolation **p** = [0.5,1, 1,1, 1] (modifying the interpolation exponent of the parameter describing the Ca^2+^-pump uptake rate).

A change in the Ca^2+^-pump uptake rate into the SR (Group 1) results in a change of the contraction times, especially for slow-twitch muscle units. To demonstrate this, different interpolation exponents *p*_1_ have been investigated, while a linear interpolation has been chosen for the remaining values of *p*_*i*_.  Figure 5 shows the results for **p** = [0.5,1, 1,1, 1] (*p*_1_ = 0.5 for the Ca^2+^-pump uptake rate into the SR and *p*_2_ = *p*_3_ = *p*_4_ = *p*_*R*_ = 1). Additionally, the results for *p*_1_ = 0.3 and *p*_1_ = 1.2 are provided in the Supplementary Material (Figures 25 and 27, resp.).

For *p*_1_ = 0.5, the curve showing contraction times versus muscle unit type exhibits a hyperbolic shape and many muscle units have short contraction times. The hyperbolic shape becomes even steeper for *p*_1_ = 0.3. For *p*_1_ = 1.2, the contraction time decreases almost linearly with increasing values of *α* and the distribution slightly shifts the contraction times toward shorter values.

**Figure 6 fig6:**
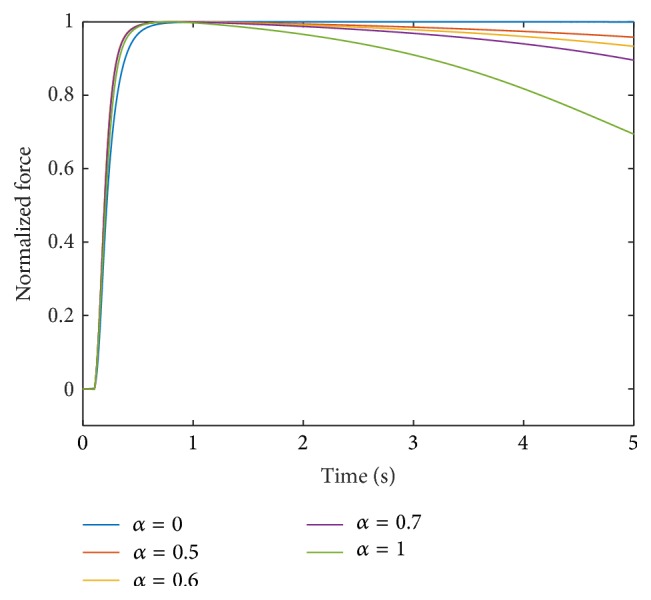
Force response at 100 Hz stimulation for different muscle units obtained by interpolating the parameter sets with **p** = [0.5,1, 1,1, 1] (modifying the interpolation exponent describing the Ca^2+^-pump uptake rate).

Comparing the results on fatigue progression in Figure 6 and Supplementary Figures 26 and 28, one can observe that the Ca^2+^-pump uptake rate does not influence the fatigue progression by much. For *p*_1_ = 0.5 the forces of muscle units with *α* = 0.5, 0.6, and 0.7 after 5000 ms of stimulation with frequency 100 Hz are 96%, 93%, and 90% of their maximal values, respectively. For *p*_1_ = 0.3, the respective forces are 95%, 93%, and 90% of their maximal forces. Moreover, an interpolation with an exponent *p*_1_ = 1.2 yields slightly higher remaining forces; that is, the forces of muscle units *α* = 0.5, 0.6, and 0.7 after 5000 ms of stimulation with frequency 100 Hz are 96%, 94%, and 91% of their maximal values, respectively.

### 3.3. Sensitivity with respect to Cross-Bridge Parameters

**Figure 7 fig7:**
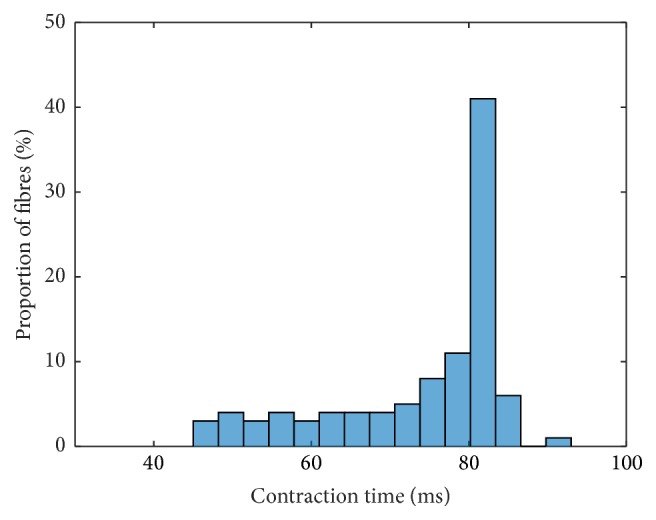
Frequency distribution of contraction times based on choosing **p** = [0.5,5, 1,1, 1] for the parameter interpolation.

**Figure 8 fig8:**
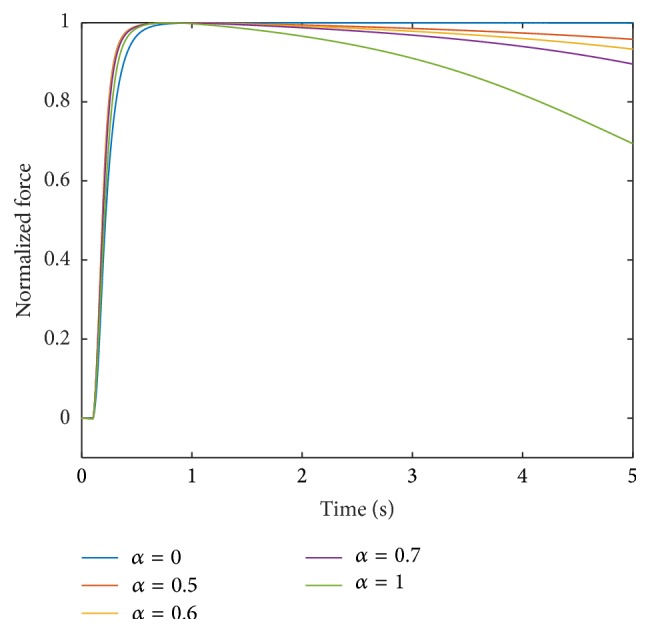
Force response at 100 Hz stimulation for different muscle units obtained by interpolating the parameter sets with **p** = [0.5,5, 1,1, 1].

Assuming independence of the parameters, the most suitable interpolation of the investigated Ca^2+^-pump uptake rate parameters provides the basis for further investigations. Next, the sensitivity due to changes in the cross-bridge parameters (Group 2) is analysed. To do so, the model was run using the interpolation exponents *p*_2_ = 0.5, *p*_2_ = 5, and *p*_2_ = 9. The interpolation of the parameters of Group 2 affects particularly the contraction times. The contraction times and force responses for different muscle units and interpolation exponent *p*_2_ = 5 are shown in Figures 7 and 8, respectively. For *p*_2_ = 5, the histogram peaks at contraction times between 80 ms and 85 ms (Figure 7). About 41% of all muscle units fall within this bin. Further results on contraction times and force responses are given for *p*_2_ = 0.5 and *p*_2_ = 9 in Figure 29/Figure 30 and Figure 31/Figure 32 within the Supplementary Material, respectively. For an interpolation exponent smaller than 1, one observes again a hyperbolic shape of the contraction times. (The reader is referred to Supplementary Figures 31 and 32, which are associated with **p** = [0.5,0.5,1, 1,1], for a representative example of a small interpolation exponent *p*_2_.) For *p*_2_ = 9 the peak in the histogram increases, and 61% of the muscle units exhibit contraction times between 80 ms and 85 ms (cf. Supplementary Figures 29 and 30).

Note that fatigue is not affected by adjusting the interpolation parameter *p*_2_, that is, by changing the interpolation scheme for the parameters associated with Group 2. For all three displayed interpolation exponents (*p*_2_ = 0.5, 5, and 9), the forces of the muscle units with *α* = 0.5, 0.6, and 0.7 are 96%, 94%, and 90% of their maximum values, respectively, after 5000 ms stimulation with frequency 100 Hz.

### 3.4. Sensitivity with respect to the RyR-Channel Rate

**Figure 9 fig9:**
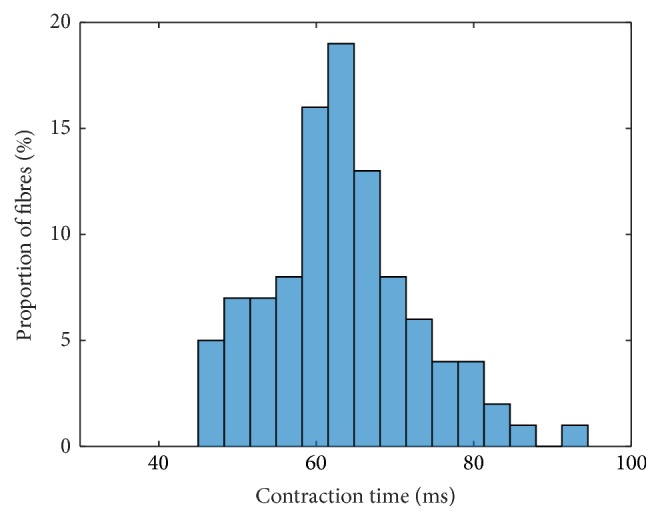
Frequency distribution of contraction times based on choosing **p** = [0.5,5, 3,1, 1] for the parameter interpolation.

**Figure 10 fig10:**
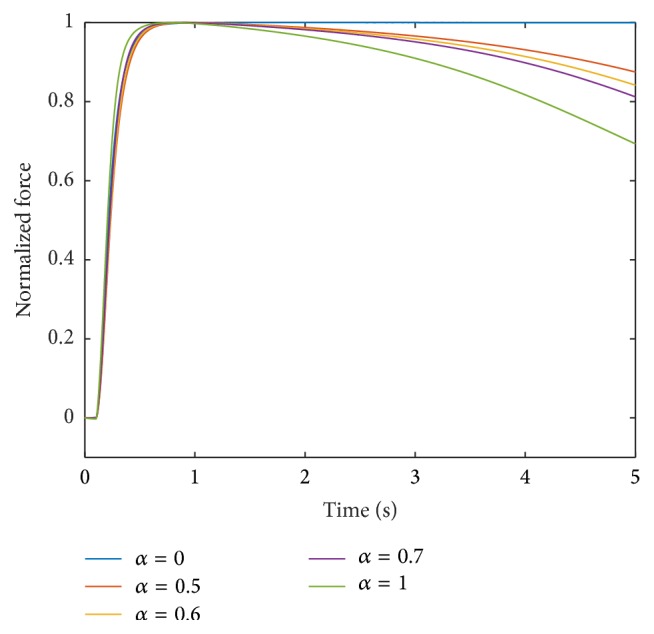
Force response at 100 Hz stimulation for different muscle units obtained by interpolating the parameter sets with **p** = [0.5,5, 3,1, 1].

For the following simulations, the exponents of the interpolation for *p*_1_, *p*_2_, and *p*_4_ are adopted from Section 3.3; that is, **p** = [0.5,5, *p*_3_, 1,1]. Now, the interpolation of the RyR-channel rate (Group 3) is considered, which impacts the contraction times and fatigue.  Figure 9/Figure 10, as well as Figure 33/Figure 34 and Figure 35/Figure 36 within the Supplementary Material, depict the histograms of the contraction times and the force responses for *p*_3_ = 2 (Figure 33/Figure 34 within the Supplementary Material), *p*_3_ = 3 (Figure 9/Figure 10), and *p*_3_ = 4 (Figure 35/Figure 36 within the Supplementary Material). For *p*_3_ = 1, the histogram shows an extreme accumulation of muscle units at contraction times of approximately 80 ms. By increasing *p*_3_, the peak of the histogram shifts toward slower muscle units and the extreme accumulation is less pronounced leading to a broader distribution. Specifically, for *p*_3_ = 2, the histogram of contraction times peaks at 70 ms. About 55% of the muscle units show contraction times between 66 ms and 73 ms (Figure 9). For *p*_3_ = 3, the peak occurs at 63 ms, for *p*_3_ = 4 the peak is broader and occurs at about 58 ms. In conclusion, one can claim that the higher the interpolation exponent *p*_3_ the shorter the most frequent contraction time.

The fatigue progression also changes with different interpolation exponents *p*_3_. For *p*_3_ = 2, the forces of the muscle units with *α* = 0.5, 0.6, and 0.7 are after 5000 ms of stimulation with 100 Hz 93%, 90%, and 86% of their maximum force, respectively. Further, for the same muscle units, *p*_3_ = 3 results in remaining forces of 88%, 84%, and 81%, while *p*_3_ = 4 results in remaining forces of 80%, 76%, and 74%, respectively.

### 3.5. Determining the Interpolation Exponent For Parvalbumin

**Figure 11 fig11:**
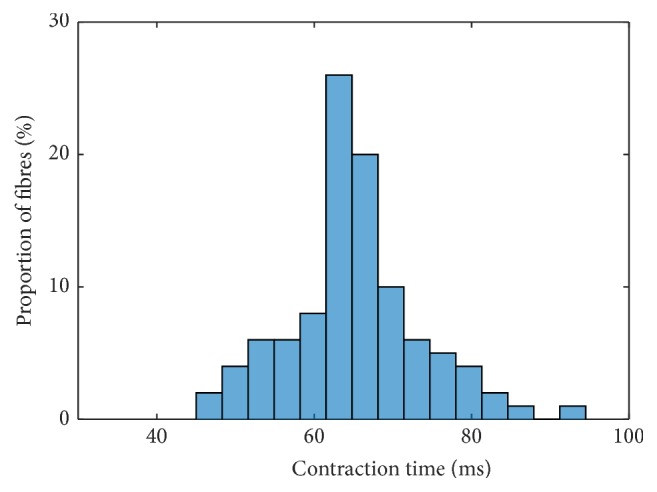
Frequency distribution of contraction times based on choosing **p** = [0.5,5, 3,4, 1] for the parameter interpolation.

**Figure 12:**
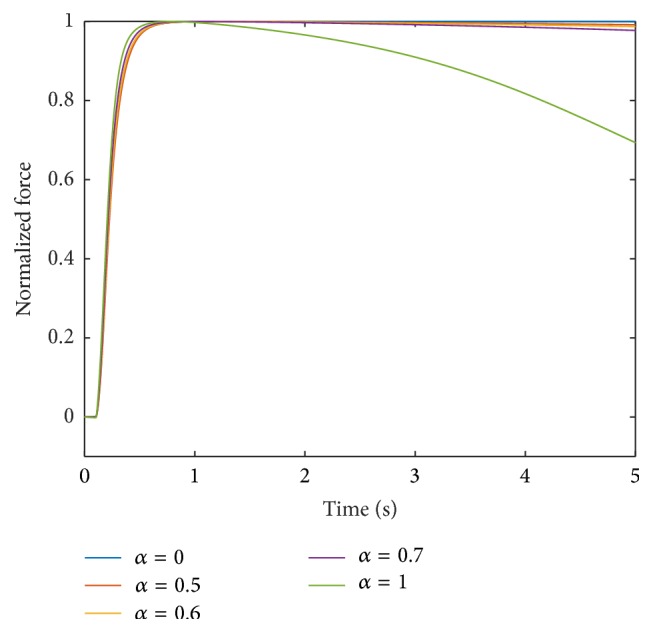
Force response at 100 Hz stimulation for different muscle units obtained by interpolating the parameter sets with **p** = [0.5,5, 3,4, 1].

As seen in the section that investigated the sensitivity with respect to parvalbumin parameters, the contraction times are not much affected by changes to the parvalbumin parameters (Group 4). This can be seen if, for example, Figure 11 is compared to Supplementary Figure 37. Following this, the interpolation of the parvalbumin parameters is used to fit experimentally determined fatigue characteristics (fatigue occurs for muscle units with *α* > 0.6). The linear interpolation of the parvalbumin parameters (*p*_4_ = 1) overestimates the fatigue progression. Hence, interpolation exponents greater than 1 are tested in order to obtain a parameter set for fast fatigable muscle units that is comparable to experimental data. For *p*_4_ = 1.5, one obtains for the muscle units with *α* = 0.5, 0.6, and 0.7 after 5000 ms of stimulation with 100 Hz forces of 94%, 91%, and 88% of their maximum values, respectively. Choosing an interpolation exponent of *p*_4_ = 2, one obtains for the same muscle units and the same stimulation protocol remaining forces of 97%, 95%, and 92% (Supplementary Figure 38). For *p*_4_ = 4 the remaining forces are 99%, 99%, and 98%, respectively (cf.  Figure 12).

**Figure 13:**
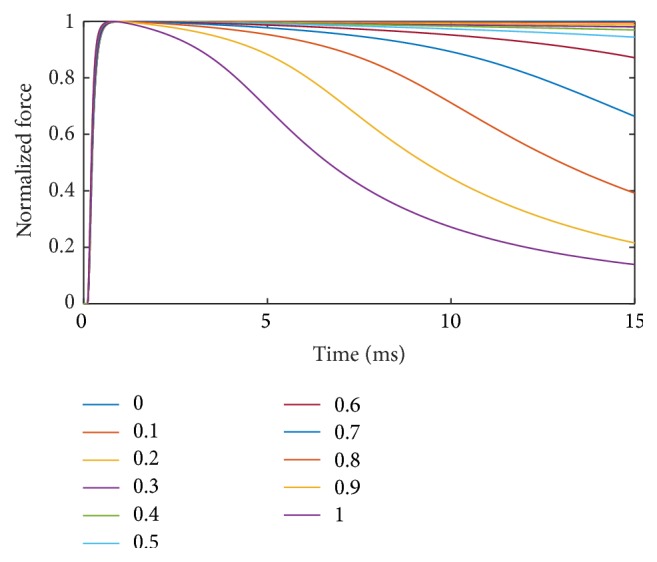
Force response at 100 Hz for different muscle units over a period of 15 s. The parameters for the different muscle units are obtained by interpolating with **p** = [0.5,5, 3,4, 1].

Up to now, all simulations were run for 5 s. If the simulations are run for 15 s, one observes that fatigue progresses over time for the muscle units with *α* > 0.5. Since this is not observed experimentally, the interpolation exponent for parvalbumin is changed to *p*_4_ = 4.  Figure 13 presents the results for 11 uniformly distributed muscle units. It can be observed that the fatigue-induced force decline for muscle units with *α* ≤ 0.5 is less than 10%. For the remaining muscle units, that is, *α* = 0.6 to *α* = 0.9, the reduction in force after 15 s covers the spread between the resulting forces of *α* = 0.5 and *α* = 1.

### 3.6. Stimulation Patterns of Burke et al. and Lannergren and Westerblad

**Figure 14:**
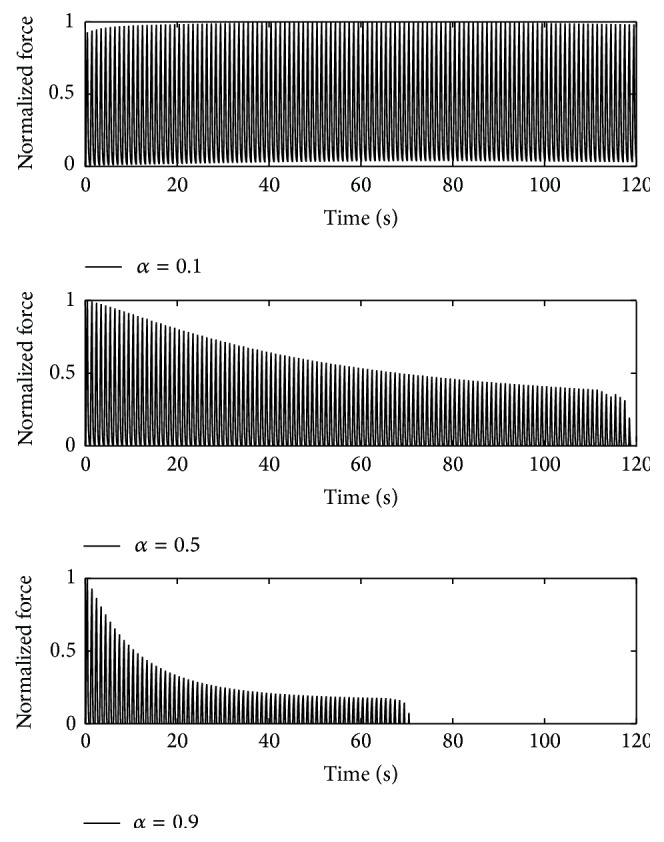
Burke et al.'s stimulation [3]: the force responses of three muscle unit types (*α* = 0.1, 0.5, and 0.9) repetitively stimulated over a period of 120 s.

**Figure 15:**
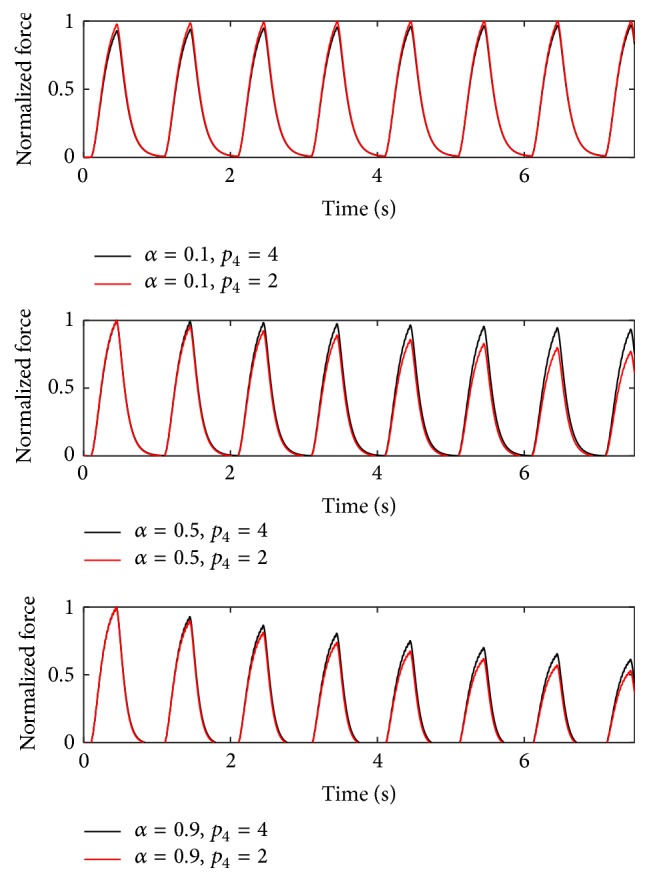
Force responses at 40 Hz stimulation for 220 ms repeated every second for different muscle units (*α* = 0.1, 0.5, and 0.9). Parvalbumin parameters *p*_4_ = 2 and *p*_4_ = 4 are considered; that is, **p** = [0.5,5, 3,2, 1] and **p** = [0.5,5, 3,4, 1].

To verify that the frequency distribution of the fatigue index can be adapted to the distribution of the short-time fatigue (remaining muscle unit forces after 5000 ms) Burke's stimulation pattern [3] is applied to the final parameter set; that is, **p** = [0.5,5, 3,4, 1]. Three muscle units (*α* = 0.1, 0.5, and 0.9) are stimulated for 120 s at 40 Hz over a time period of 330 ms. This is repeated every second. (No stimulation is applied in the remaining 670 ms of each second.) The results are presented in Figures 14 and 15.

In Figure 15, the force response in the first 7.5 s can be seen for two different parvalbumin interpolation exponents; that is, *p*_4_ = 2 and *p*_4_ = 4. The solid line shows the force response for *p*_4_ = 4; the dashed line is simulated with *p*_2_ = 2. For muscle unit types 0.5 and 0.9, the force declines for *p*_2_ = 2 more rapidly than for *p*_4_ = 4. Muscle unit type 0.1 shows no fatigue in either case.

The muscle unit with *α* = 0.1 shows no fatigue within the interval. Hence, Burke's fatigue index for this muscle unit is 1. The force response of the muscle unit with *α* = 0.5 declines approximately linearly until 118 s. The second last force peak is considerably lower than the previous ones. The last force peak is missing entirely. The muscle unit with *α* = 0.9 shows an exponential decline in the force response in the first 40 s, before settling at a force of about 20% of its maximum value. After 68 s stimulation time, the force decreases rapidly such that no further force peaks can be observed any more after 72 s. Following the nomenclature of [3], this results in a fatigue index of 0 for the muscle units with *α* = 0.5 and 0.9.

**Figure 16:**
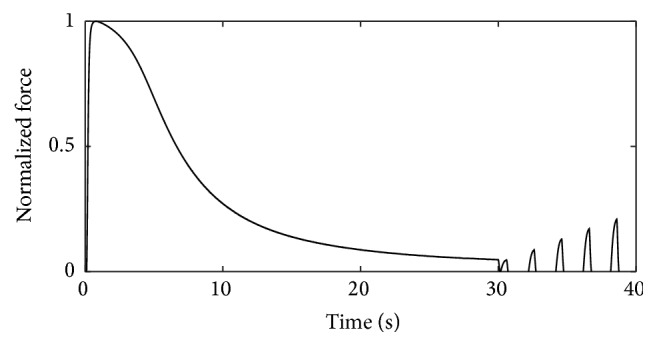
Lannergren and Westerblad's stimulation pattern [23]. Force response for a fast-twitch muscle unit (*α* = 1) for a tetanic stimulation followed by a stimulation at 100 Hz for 500 ms repeated every two seconds.

Figure 16 shows the force response to a stimulation pattern similar to that presented by Lannergren and Westerblad [23]. The fastest muscle unit (*α* = 1) is first continuously stimulated with a frequency of 100 Hz for 30 s, followed by a Burke-like stimulation, that is, stimulating every two seconds for 500 ms with 100 Hz.

The force declines in the first 30 s to 5% of the maximum force. The subsequent stimulation with resting time results in a regeneration of the force. The peak of the 5th train of stimuli shows already 20% of the maximum force. This recovery is qualitatively consistent with experimental data; however, quantitatively, the recovery is not as fast as in experiments.

## 4. Discussion

Before discussing the modelling assumptions and the results in a broader context, the findings of the interpolation study are summarized in the following. Note that for *p*_*i*_ > 1 the interpolated values remain for a wider range of motor units closer to the slow-twitch value, before accelerating to the fast-twitch value. For *p*_*i*_ < 1, the behaviour is just the opposite.

- If the binding and dissociation rates to and from parvalbumin are not significantly reduced, intermediate muscle units show more fatigue than the fastest muscle unit (cf.  Figure 3 and Supplementary Figures 17, 19, 21, and 23). Such behaviour has not been observed experimentally.
- Increasing (decreasing) the interpolation coefficient for the calcium pump uptake rate, *p*_1_, leads to a shift toward fast (slow) contraction times (cf.  Figure 5 and Supplementary Figures 25 and 27).
- Values of the interpolation coefficient for the reaction rates of the cross-bridge dynamics model, *p*_2_, greater than one lead to a peak in the contraction times histogram at long contraction times, and the height of the peak increases with *p*_2_. Values of *p*_2_ smaller than one yield a shift of the distribution toward slow contraction times (cf. Figure 7 and Supplementary Figures 29 and 31).
- The interpolations of both the calcium pump uptake rate and the cross-bridge dynamics reaction rates hardly affect fatigue.
- By increasing the interpolation coefficient of the RyR-channel rate, *p*_3_, the peak of the contraction times histogram shifts toward slower muscle units and it is less pronounced leading to a broader distribution (cf.  Figure 9 and Supplementary Figures 33 and 35). In conclusion, one can claim that the higher the interpolation exponent *p*_3_, the shorter the most frequent contraction time.
- Increasing the interpolation coefficients of the RyR-channel rate, *p*_3_, results in faster fatiguing intermediate muscle units.
- Increasing (decreasing) the interpolation coefficient of the parvalbumin parameters, *p*_4_, leads to weaker (stronger) fatigue progression of intermediate muscle units (cf. Figures 12 and 13 and Supplementary Figure 38).
- Changes in the interpolation coefficient of the parvalbumin parameters, *p*_4_, do not affect the contraction times (cf. Figure 11 and Supplementary Figure 37).

Although muscle fatigue has been well studied in experiments on whole muscles and single intact and stripped fibres, the cellular processes leading to muscle unit-dependent (peripheral) muscle fatigue are still not entirely understood. Due to the fact that the Shorten et al. model [14] closely reflects the physiological processes leading from electrical excitation to contraction in skeletal muscle fibres and also contains a description of different biochemical processes leading to muscle fatigue, it can be used to generate data that are currently not available from the literature. In the present study, this model has been extended to a description of the different muscle units in a muscle, which are subject to different levels of fatigue. To this end, the parameters of the slow-twitch and fast-twitch versions of the Shorten et al. model [14] have been modified and interpolated using smooth and continuous interpolation functions. While the model is capable of closely matching experimental data, one should mention that, in physiology, some properties might also arise only after a threshold or are grouped into discrete classes. One could certainly consider within this work also step functions and, hence, probably even further improve the results. This, however, leads to further degrees of freedom that make it even harder to investigate and analyse the results.

Continuous distributions of the functional properties (e.g., contraction time, maximum shortening velocity, or the activity of the mitochondrial enzymes such as SDH) across the muscle units of skeletal muscles are well established (cf., e.g., [10, 13] and references therein). In contrast, continuous distributions of biophysical parameters, as assumed in this work, have not been described in the experimental literature. Bottinelli and Reggiani [13] distinguish two main mechanisms leading to the heterogeneity among the functional properties of muscle units. First, the heterogeneity can result from different isoforms that exist for many muscle proteins. This does not contradict the assumption of continuous distributions of biophysical parameters, since muscle fibres can coexpress different isoforms. For example, different isoforms of the MHC have been found to coexist in one muscle fibre type [10]. Second, the same gene can be expressed differently, depending on factors such as neural activation, mechanical load, or hormones. For example, the density of SR Ca^2+^ pumps is much higher in fast-twitch than in slow-twitch muscle fibres [7]. It can be easily imagined that the up- and downregulation of genes yield a continuous distribution of the biophysical properties.

Although closely resembling the physiological processes of the ECC, the model of Shorten et al. [14] does not contain a description of the cell metabolism. In detail, while Shorten et al. model the accumulation of ADP and inorganic phosphate in the myoplasm, there are unlimited amounts of ATP available within the cells. The depletion of ATP and the accumulation of ADP and inorganic phosphate in the myoplasm are known to affect the force generating capability of muscle fibres [6]. Especially for analysing sustained contractions, the model should be extended by a more detailed description of the cell metabolism that accounts, for example, for the ATP generation in the mitochondria and the supply of oxygen and nutrients to the cell. This however is beyond the scope of this work. Including a description of the cell metabolism within the model would affect the fatigue progression during sustained contractions as considered in Figures 13, 14, 15, and 16. For now, the predicted fatigue is mainly influenced by the RyR-channel rate and the parvalbumin dynamics (binding and dissociation rates of calcium and magnesium ions to and from parvalbumin). Including the cell metabolism as a third mechanism contributing to fatigue might require a reparametrisation of the RyR-channel rate and the parvalbumin dynamics. In experimental conditions, it is difficult to separate the influence of different factors contributing to fatigue. A biophysical muscle model that includes a description of the cell metabolism could support the experimentalist in this task.

As a model can only be as good as the data to which it is fitted, it is important to identify and focus in experimental studies on those parameters that exhibit the most sensitivity with respect to muscle fatigue. The resulting model produces fast fatigable and slow fatigue-resistant muscle units. What is not well realised yet is the fast fatigue-resistant muscle unit type. This muscle unit type is characterised by a fast contraction (comparable with the fast fatigable muscle unit) and little fatigue. Within the presented model, the fastest muscle unit that shows only little fatigue (*α* = 0.5) contracts in about 60 ms, which is 150% of the contraction time of the fastest muscle unit. In general, however, the contraction times could be improved by modifying the cross-bridge parameters for fast-twitch muscle units such that the proposed model would exhibit contraction times also below 40 ms. In [11], for example, a contraction time of 30 ms was assumed for the fastest muscle unit. In the present work, the cross-bridge parameters of Shorten et al. [14] have been scaled by a factor of 1/10 (cf. [18]), to account for slower kinetics and longer muscle fibres in humans compared to mice [21]. This scaling yielded contraction times approximately between 40 ms and 90 ms. A larger scaling factor, for example, 1/5, probably leads to better results. The aim of this study, however, is not to identify an optimal set of parameters but to investigate the sensitivity of the model to variations in the parameters, in order to reach a conclusion on their importance with respect to muscle fatigue.

Besides the contraction times, the reaction rates of the cross-bridge cycle determine the shape of the single twitch of a muscle fibre. The parameter set suggested by Razumova et al. for their cross-bridge dynamics model [18] (which is used within the model of Shorten et al. [14]) predicts twitch shapes that agree well with experimentally measured ones. A consistent scaling of the entire parameter set of the cross-bridge dynamics model of Razumova et al. [18] leads to twitches with different time course but similar shape. For example, Shorten et al. [14] successfully modelled twitches and tetanic contractions of soleus and EDL muscles of mice by multiplying the entire parameter set of Razumova et al. by factors of 10 and 30, respectively. In accordance with this, we grouped together the cross-bridge parameters within one group, such that the resulting model predicts twitch shapes that are scaled versions of the ones predicted by the Razumova et al. model.

Two isoforms, namely, SERCA2 in slow-twitch fibres and SERCA1 in fast-twitch fibres, exist for the SR Ca^2+^ pump in skeletal muscle, and the density of pumps is much higher in fast-twitch than in slow-twitch fibres (cf. [7]). Following this, the SR Ca^2+^ pumping rate was treated as an individual parameter group in our model. Moreover, since there exist differences in the kinetics of the RyR between slow-twitch and fast-twitch muscle fibres [13], the RyR Ca^2+^ current is also treated as an individual parameter set.

Finally, we investigated the model behaviour for different interpolations of the binding and dissociation rates to and from parvalbumin. Since parvalbumin is only present in fast-twitch fibres [22], the corresponding rates equal zero in the slow-twitch version of the model. We obtained realistic results for an interpolation coefficient of *p*_4_ = 4, which means that, for the majority of muscle units, the parvalbumin parameters are similar to the slow-twitch model (i.e., close to zero), and parvalbumin only affects few (fast-twitch) muscle units. This finding corresponds well with the fact that there are typically many slow-twitch and few fast-twitch muscle units in a muscle [11]. Further, for the parameter interpolation, we grouped together the binding and dissociation rates of calcium and magnesium ions to and from parvalbumin into one group. Another possibility would have been to treat each of the binding and dissociation rates independently. This, however, would further increase the model complexity. Moreover, there is no experimental data available to justify a certain choice over another.

The outcome of stimulating the fastest muscle unit with a long-term tetanic stimulation protocol with respect to fatigue fits well the experimental data presented in [23]. The progression of the other muscle units cannot be reviewed as there is no experimental data available. The fatigue of intermediate muscle units was designed such that only 30% of the muscle units show fatigue. This is in agreement with the experimental data presented in [29]. The parameters, however, could be further improved given further data. In this work, the fatigue of different muscle units was adapted to the frequency distribution of fatigue indices following [3]. To confirm this approach, it was expedient to use a stimulation protocol based on the pattern proposed in [3]. The results for three different muscle unit types (*α* = 0.1, 0.5, and 0.9) over a period of 120 s can be seen in Figure 14. The presented simulation shows that the parameters could still be improved by fitting them to new experimental data as they become available. However, it was not the objective of this work to provide a perfect set of parameters to model a specific muscle but rather to reveal the sensitivity of the model response to changes in the parameters.

Applying Lannergren and Westerblad's stimulation pattern [23] to a fast-twitch muscle fibre, the model predicted a slow recovery. In the experimental data presented in [23], the muscle fibre recovers faster; that is, it produces more force in the stimulation pulses after the tetanic stimulation. To mimic this behaviour, other states than the force output should be investigated to analyse which processes influence this recovery. This, however, was not the focus of this study. The key advantage of employing a biophysical model such as the one presented within this work, however, is that one can easily investigate other quantities than the force, for example, the intracellular Ca^2+^ concentration.

Additionally to the investigated characteristics, that is, fatigue and contraction times, the model could be examined regarding the Ca^2+^ concentration progression as there exist more experimental data; see, for example, [22]. While the authors in [14] also determined the action potential conduction velocities on the fatigued and nonfatigued muscle fibres, muscle unit-specific experimental data are rare [30, 31]. In [32], for example, only conduction velocities for slow-twitch and fast-twitch muscle are shown.

## 5. Summary and Outlook

This work presents an extension of the Shorten et al. model [14], which has been experimentally validated for slow-twitch and fast-twitch muscles, to investigate physiological properties of the different muscle units, which do exist in human skeletal muscles. To this end, the parameters of the computational model are determined by interpolating the parameter sets of the slow-twitch and fast-twitch muscles in different ways. The model revealed that different interpolations are required for different parameter groups to match experimental results. Using the resulting model, we identified one parameter group that predominantly affects fatigue, namely, the parvalbumin parameters, and two parameter groups that affect predominantly the contraction times, namely, the Ca^2+^ channel uptake rate and the cross-bridge parameters. Moreover, we found that the RyR current affected both contraction time and fatigue. Due to the fact that the model closely resembles the underlying physiological processes, it could potentially support the experimentalist investigating the mechanisms leading to peripheral muscle fatigue in the different muscle units. In turn, additional muscle unit-specific experimental data can improve mathematical models and hence contribute to an improved and validated mathematical model of the muscle units to investigate central fatigue using detailed chemoelectromechanical models of the entire neuromuscular system [21, 33–36].

## Supplementary Material

Force responses and contraction time histograms resulting from simulations with additional combinations of interpolation exponents.

## Figures and Tables

**Table 1:** Parameter groups.

Group	Parameter	Definition
1	61	SR Ca^2+^-pump uptake rate

2	89	Rate of cross-bridge attachment
	90	Rate of pre-power stroke cross-bridge detachment
	91	Forward rate of the power stroke
	92	Reverse rate of the power stroke
	93	Rate of post-power stroke cross-bridge detachment
	94	Rate of myoplasmic phosphate degradation

3	99	RyR Ca^2+^ current

4	71, 72	Binding and dissociation rate of Ca^2+^-parvalbumin
	74, 75	Binding and dissociation rate of Mg^2+^-parvalbumin

*R*	1	Membrane capacitance
	2	Ratio of t-tubule membrane area to sarcolemma membrane area
	8, 9	t-tubule diffusion time constant
	10	Fraction of fibre occupied by t-tubules
	11, 12	Interstitial space diffusion time constant
	13, 14	Resting K^+^ and Na^+^ current
	25, 28	Factors for slow inactivation Na^+^-gating
	38, 39, 40	Cl^−^, K^+^, and Na^+^ conductance in two compartment model
	41	Maximum IR conductance
	48	Maximum Na^+^-K^+^ pump activity
	63	SR Ca^2+^ leak constant
	68	Binding rate of Ca^2+^-troponin

Group 1 contains the SR Ca^2+^-pump uptake rate parameter that is associated with force reduction; Group 2 contains the parameters associated with the cross-bridge cycle; Group 3 contains the parameter associated with rate of Ca^2+^ release from the SR; Group 4 describes parameters associated with the binding to and the dissociation from parvalbumin; Group *R* contains the remaining parameters that influence the muscle unit type behaviour within the model.

**Table 2:** Relative errors in the fatigue progression (*E*_*f*_) and the contraction time (*E*_CT_).

*p*_1_	*p*_2_	*p*_3_	*p*_4_	*p*_*R*_	*E*_*f*_	*E*_CT_	Remark
1	1	1	1	1	0.9548	2.0474	*∗*
2	2	2	2	2	1.0898	1.5683	*∗*
5	5	5	5	5	0.7774	2.2112	*∗*
0.5	0.5	0.5	0.5	0.5	0.8572	2.9541	*∗*
1	1	1	1	1	0.1201	0.9114	
0.5	1	1	1	1	0.1309	1.1356	
0.3	1	1	1	1	0.1364	1.3900	
1.2	1	1	1	1	0.1166	0.9038	
0.5	5	1	1	1	0.3881	1.4458	
0.5	9	1	1	1	0.1273	1.9306	
0.5	0.5	1	1	1	0.1312	1.8316	
0.5	3	1	1	1	0.3911	0.9774	
0.5	5	2	1	1	0.1811	0.3267	
0.5	5	3	1	1	0.2757	0.4990	
0.5	5	4	1	1	0.3990	0.8035	
0.5	5	3	1.5	1	0.1606	0.4371	
0.5	5	3	2	1	0.1012	0.3076	
0.5	5	3	4	1	0.0288	0.2484	

*p*_1_–*p*_4_ are the interpolation coefficients for Groups 1–4, and *p*_*R*_ is the interpolation coefficient for the remaining parameters.

^*∗*^Results before scaling the parameters in the parvalbumin group (Group 4) by 1/20. See text for details.
