# Supplementary material for: The Role of Parvalbumin, Sarcoplasmatic Reticulum Calcium Pump Rate, Rates of Cross-Bridge Dynamics, and Ryanodine Receptor Calcium Current on Peripheral Muscle Fatigue: A Simulation Study
Source: Comput Math Methods Med. 2016 Oct 20;2016:3180205. doi: 10.1155/2016/3180205 (PMC5131563; doi:10.1155/2016/3180205)

The Role of Parvalbumin, Sarcoplasmatic Reticulum Calcium Pump Rate, Rates of Cross-Bridge Dynamics, and Ryanodine Receptor Calcium Current on Peripheral Muscle Fatigue: a Simulation Study (Supplemental Material)

Oliver Röhrle[1,2,*], Verena Neumann[1], Thomas Heidlauf[1,2]

1 Institute of Applied Mechanics (CE), University of Stuttgart, Stuttgart, Germany
2 Stuttgart Research Centre for Simulation Technology (SimTech), University of Stuttgart, Stuttgart, Germany

* **Corresponding Author:**
Oliver Röhrle, Institute of Applied Mechanics (CE)
Pfaffenwaldring 7, University of Stuttgart, 70569 Stuttgart, Germany
Email: roehrle@simtech.uni-stuttgart.de
Phone: +49 711 684 66284

Supplemental Material

Uniform Interpolation

The contraction times resulting from linearly interpolating the parameters for the slow-twitch muscle unit and the parameters for the fast-twitch muscle unit are shown in Fig. 1. It can be observed that the contraction times are distributed in a hyperbolic form in the interval between the fastest and the slowest muscle unit type, ranging from 38 ms to 91 ms. About 50 % of the muscle units are in the lower third in the interval of all exhibited contraction times. The fatigue results can be seen in Fig. 2. The intermediate muscle units do not show intermediate fatigue instead they show more fatigue than muscle unit 1. The force decline due to fatigue occurs too early and is too steep. Furthermore, there are too many muscle units exhibiting fatigue compared to the portion showing fatigue in experimental data (recall that less than 40 % show fatigue).

Next, perturbations of the interpolation exponent are considered by choosing for all values of p_i equal to either 2, 5, or 0.5. The results can be seen in Fig. 3, Fig. 4, Fig. 5, Fig. 6, Fig. 7 and Fig. 8, respectively.

For a quadratic interpolation ($\boldsymbol{p} = [2, 2, 2, 2, 2]$) the contraction times are similarly to those obtained from linear interpolation. The only notable difference can be observed within the slowest 10 % of the muscle units, which have contraction times of about 90 ms. The fatigue progression, however, differs if compared to the results obtained using a linear interpolation. For example, muscle unit type 0.5 shows a later force decline. For $\boldsymbol{p} = [5, 5, 5, 5, 5]$ 40 % of the muscle units exhibit non-physiological contraction times (about 90 ms) while the rest is equally distributed. For muscle unit type 0.5, there is almost no observable fatigue. Further, muscle unit type 0.6 has a residual force after 5000 ms that is less than the desired one for muscle unit type 1. Muscle unit type 0.7 shows even more fatigue. Moreover, muscle unit type 1 shows less fatigue than the intermediate muscle units. Choosing interpolation exponents smaller than 1, e. g. $\boldsymbol{p} = [0.5, 0.5, 0.5, 0.5, 0.5]$, the hyperbolic form of the contraction time distribution is more pronounced and fatigue of intermediate muscle units approach the fatigue progression of muscle unit type 1.

Fig. 1 Histogram resulting from the distribution of contraction times. Linear interpolation $\boldsymbol{p} = [1, 1, 1, 1, 1]$: For linear interpolated parameters the contraction times are distributed in a hyperbolic form.

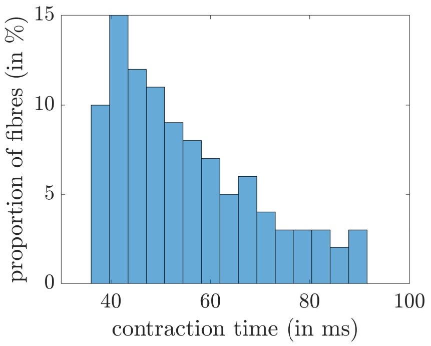

Fig. 2 Force responses at 100 Hz stimulation for different muscle unit types. Linear interpolation $\boldsymbol{p} = [1, 1, 1, 1, 1]$: The fatigue occurs early and heavily.

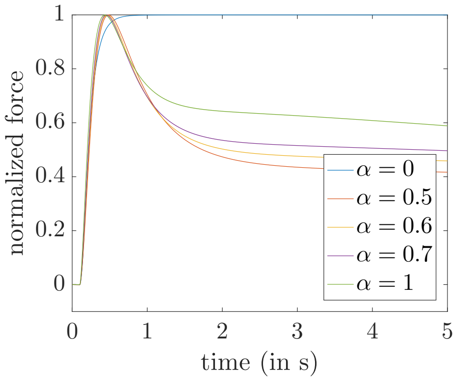

Fig. 3 Histogram resulting from the distribution of contraction times using for the parameter interpolation the exponents $\boldsymbol{p} = [2, 2, 2, 2, 2]$

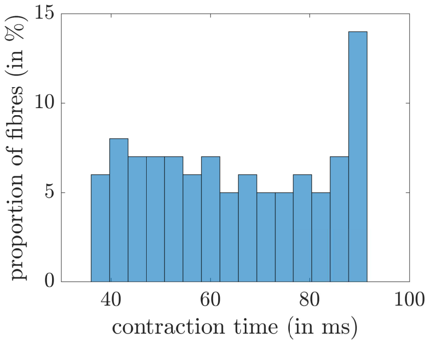

Fig. 4 Force responses at 100 Hz stimulation for different muscle unit types using for the parameter interpolation the exponents $\boldsymbol{p} = [2, 2, 2, 2, 2]$

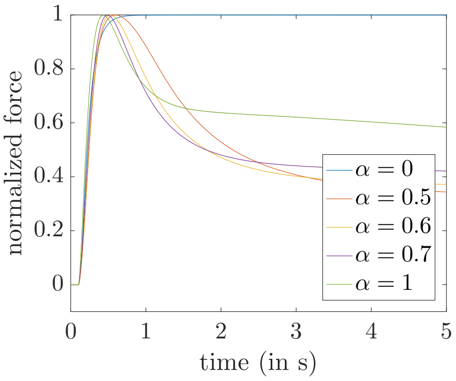

Fig. 5 Histogram resulting from the distribution of contraction times using for the parameter interpolation the exponents $\boldsymbol{p} = [5, 5, 5, 5, 5]$

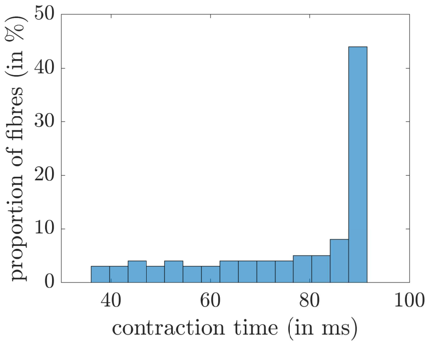

Fig. 6 Force responses at 100 Hz stimulation for different muscle unit types using for the parameter interpolation the exponents $\boldsymbol{p} = [5, 5, 5, 5, 5]$

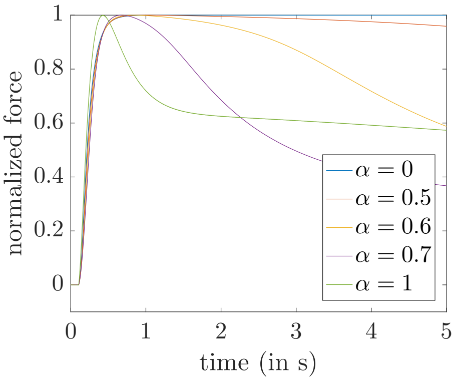

Fig. 7 Histogram resulting from the distribution of contraction times using for the parameter interpolation the exponents $\boldsymbol{p} = [0.5, 0.5, 0.5, 0.5, 0.5]$

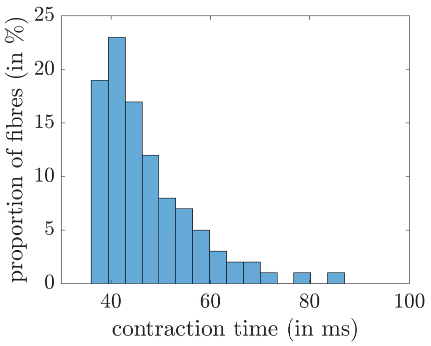

Fig. 8 Force responses at 100 Hz stimulation for different muscle unit types using for the parameter interpolation the exponents $\boldsymbol{p} = [0.5, 0.5, 0.5, 0.5, 0.5]$

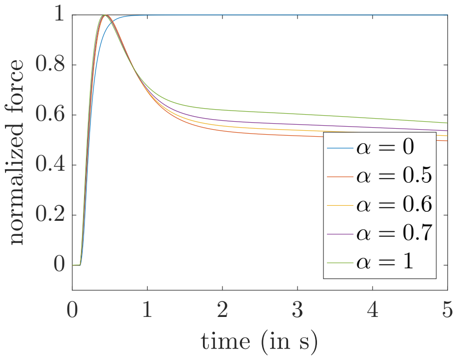

One can clearly see that the contraction time and fatigue are substantially influenced by the choice of interpolation. Furthermore, these results obtained through uniform interpolation underpin the need for introducing different interpolation coefficients for different parameter groups.

Group 1 – Calcium Pump Uptake Rate

The following figures (Fig. 9, Fig. 10, Fig. 11, and Fig. 12) show the histograms and the force responses if the parameters of Group 1 are interpolated with $p_1 = 0.3$ and $p_1 = 1.2$, while the remaining groups are linearly interpolated, i. e., $p_2 = p_3 = p_4 = 1$.

Fig. 9 Histogram resulting from the distribution of contraction times using for the parameter interpolation the exponents $\boldsymbol{p} = [0.3, 1, 1, 1, 1]$

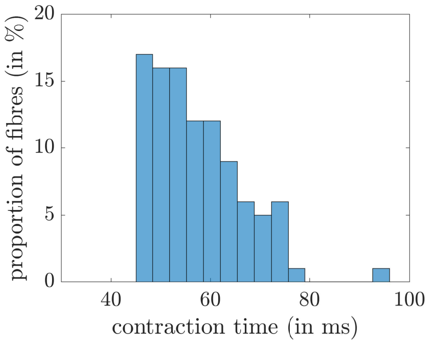

Fig. 10 Force responses at 100 Hz stimulation for different muscle unit types using for the parameter interpolation the exponents $\boldsymbol{p} = [0.3, 1, 1, 1, 1]$

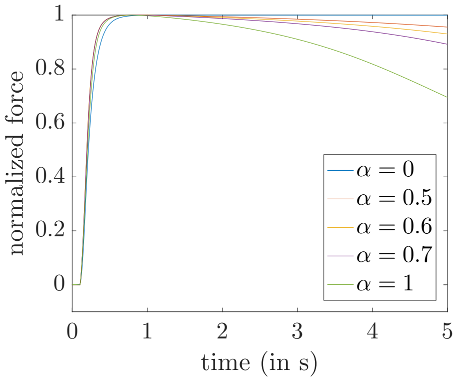

Fig. 11 Histogram resulting from the distribution of contraction times using for the parameter interpolation the exponents $\boldsymbol{p} = [1.2, 1, 1, 1, 1]$

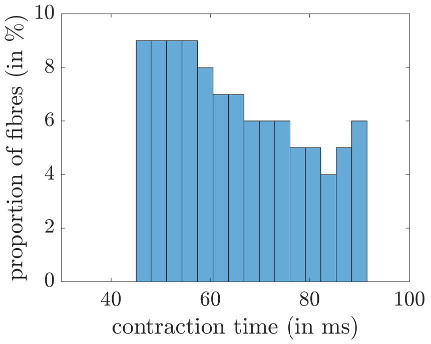

Fig. 12 Force responses at 100 Hz stimulation for different muscle unit types using for the parameter interpolation the exponents $\boldsymbol{p} = [1.2, 1, 1, 1, 1]$

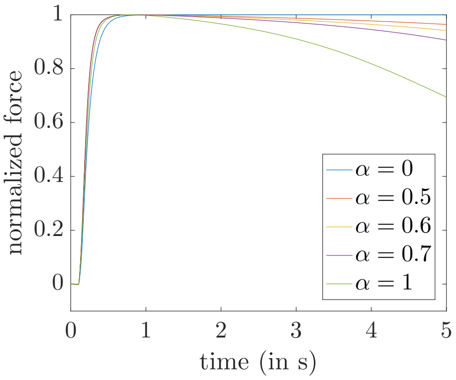

Group 2 – Cross-Bridge Parameters

Fig. 13, Fig. 14, Fig. 15, and Fig. 16 provide additional results on contraction times and force responses if the parameters associated with Group 2, i.e., all parameters associated with the cross-bridge dynamics, are interpolated with $p_2 = 9$ (Fig. 13 and Fig. 14) and $p_2 = 0.5$ (Fig. 15 and Fig. 16). The results obtained for these two interpolation exponents demonstrate that the interpolation of the cross-bridge parameters has, as expected, a significant effect on the contraction times but hardly any effect on the fatigue progression.

Fig. 13 Histogram resulting from the distribution of contraction times using for the parameter interpolation the exponents $\boldsymbol{p} = [0.5, 9, 1, 1, 1]$

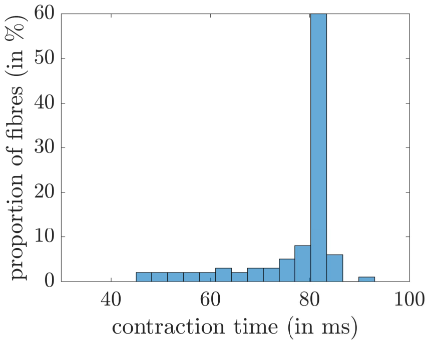

Fig. 14 Force responses at 100 Hz stimulation for different muscle unit types using for the parameter interpolation the exponents $\boldsymbol{p} = [0.5, 9, 1, 1, 1]$

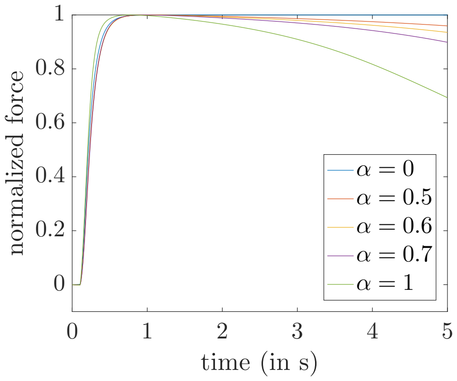

Fig. 15 Histogram resulting from the distribution of contraction times using for the parameter interpolation the exponents $\boldsymbol{p} = [0.5, 0.5, 1, 1, 1]$

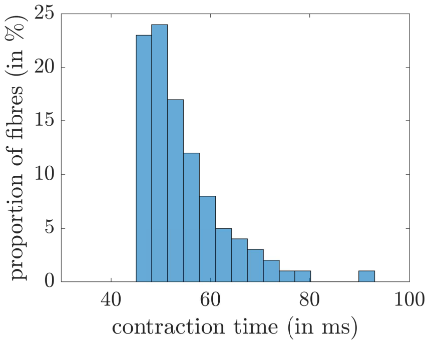

Group 3 – RyR-Channel Rate Parameters

Fig. 17, Fig. 18, Fig. 19, and Fig. 20 provide additional results on contraction times and force responses for interpolating the parameters of Group 3 with $p_3 = 2$ and $p_3 = 4$.

Fig. 16 Force responses at 100 Hz stimulation for different muscle unit types using for the parameter interpolation the exponents $\boldsymbol{p} = [0.5, 0.5, 1, 1, 1]$

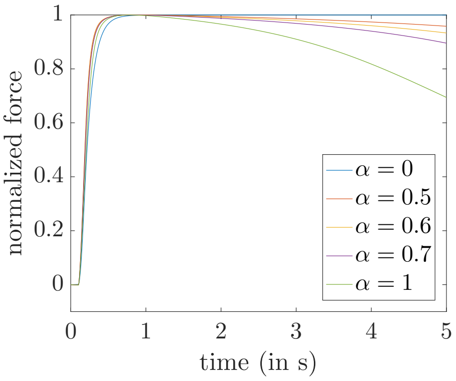

Fig. 17 Histogram resulting from the distribution of contraction times using for the parameter interpolation the exponents $\boldsymbol{p} = [0.5, 5, 2, 1, 1]$

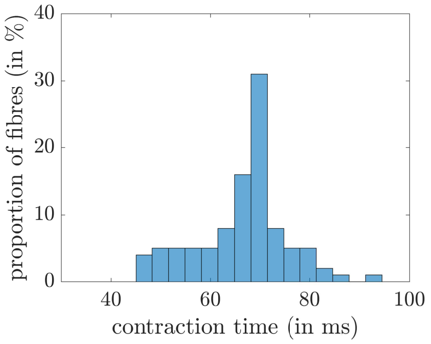

Group 4 – Parvalbumin Parameters

Fig. 21, and Fig. 22 provide additional results on contraction times and force responses for interpolating the parameters of Group 4 with $p_4 = 2$.

Fig. 18 Force responses at 100 Hz stimulation for different muscle unit types using for the parameter interpolation the exponents $\boldsymbol{p} = [0.5, 5, 2, 1, 1]$

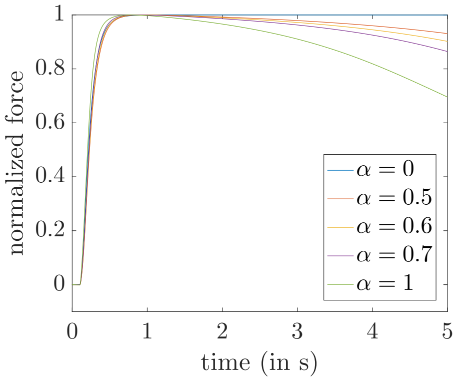

Fig. 19 Histogram resulting from the distribution of contraction times using for the parameter interpolation the exponents $\boldsymbol{p} = [0.5, 5, 4, 1, 1]$

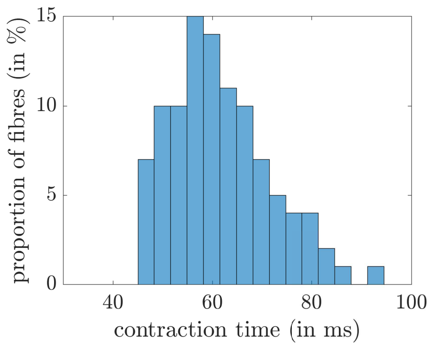

Fig. 20 Force responses at 100 Hz stimulation for different muscle unit types using for the parameter interpolation the exponents $\boldsymbol{p} = [0.5, 5, 4, 1, 1]$

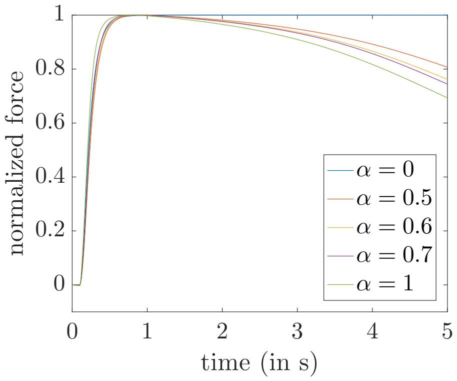

Fig. 21 Histogram resulting from the distribution of contraction times using for the parameter interpolation the exponents $\boldsymbol{p} = [0.5, 5, 3, 2, 1]$

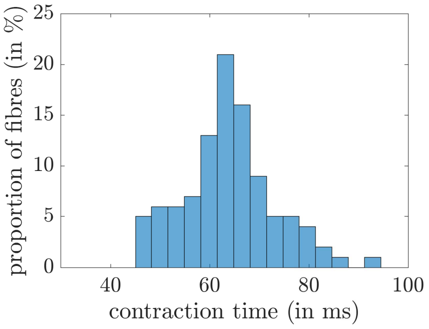

Fig. 22 Force responses at 100 Hz stimulation for different muscle unit types using for the parameter interpolation the exponents $\boldsymbol{p} = [0.5, 5, 3, 2, 1]$

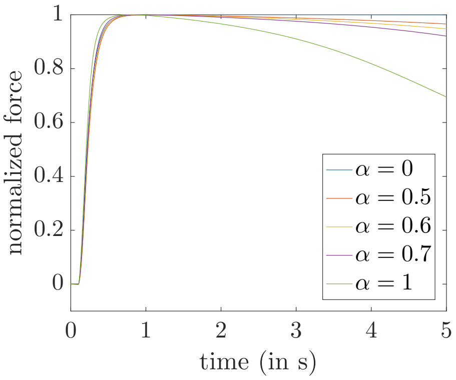

Supplement: Supplementary file 1 — Force responses and contraction time histograms resulting from simulations with additional combinations of interpolation exponents. [file 3180205.f1.pdf]
